# Upregulation of Intestinal Barrier Function in Mice with DSS-Induced Colitis by a Defined Bacterial Consortium Is Associated with Expansion of IL-17A Producing Gamma Delta T Cells

**DOI:** 10.3389/fimmu.2017.00824

**Published:** 2017-07-12

**Authors:** Ming Li, Bing Wang, Xiaotong Sun, Yan Tang, Xiaoqing Wei, Biying Ge, Yawei Tang, Ying Deng, Chunyang He, Jieli Yuan, Xia Li

**Affiliations:** ^1^Department of Microecology, College of Basic Medical Science, Dalian Medical University, Dalian, China; ^2^Department of Immunology, College of Basic Medical Science, Dalian Medical University, Dalian, China; ^3^The Core Laboratory of Medical Molecular Biology of Liaoning Province, Dalian Medical University, Dalian, China; ^4^Functional Laboratory, College of Basic Medical Science, Dalian Medical University, Dalian, China

**Keywords:** inflammatory bowel disease, dysbiosis, bacterial consortium transplantation, γδT cells, IL-17A, occludin, intestinal barrier function

## Abstract

Bacterial consortium transplantation (BCT) is a promising alternative to fecal microbiota transplantation in treating inflammatory bowel disease (IBD). Here, we showed that a defined bacterial consortium derived from healthy mice was able to enhance the intestinal barrier function of mice with dextran sulfate sodium (DSS)-induced colitis. Interestingly, we found that the bacterial consortium significantly promoted the expansion of IL-17A-producing γδT (γδT17) cells in colonic lamina propria, which was closely associated with changing of intestinal microbial composition. The increased IL-17A secretion upon treatment with microbial products derived from the bacterial consortium was accompanied with upregulation of TLR2 expression by γδT cells, and it might be responsible for the upregulation of mucosal barrier function through IL-17R-ACT1-mediated recovery of the disrupted occludin subcellular location. Changing of some specific microbial groups such as *Bifidobacterium* and *Bacillus* spp. was closely correlated with the promotion of TLR2^+^ γδT cells. Our results support that BCT can restore the alliance between commensal microbiota and intestinal γδT cells, which contributes to the improvement of intestinal barrier function. This study provides new insight into the development of bacteria transplantation therapy for the treatment of IBD.

## Introduction

The gastrointestinal (GI) illness inflammatory bowel disease (IBD) is characterized by chronic intestinal inflammation caused by immune responses against the patient’s own organs. Several factors are found to play important roles in the development and progression of IBD, including host genotype, the composition of intestinal microbiota, immune disequilibrium, as well as environmental factors (1, 2). In recent years, intestinal dysbiosis has been increasingly linked to IBD (3, 4). Current research suggests that disruption of the alliance between the host immune system and commensal microbiota may contribute to the pathogenesis and status of IBD (5).

Many studies have supported the concept that disrupted intestinal microbiota may be a contributing factor in patients with IBD, which can be initiated because of a dysregulated epithelial-immune cell communication (6). The appreciation of commensal microbes as being beneficial for the host, especially in shaping the intestinal barrier, has been the justification for using fecal microbiota transplantation (FMT) therapy for the treatment of IBD (7). FMT has resulted in disease remission and improved quality of life (8). However, some unfavorable outcomes of FMT were also reported (9). Colonization of transplanted bacteria in the GI tract was found to differ significantly among patients (10). In addition, the safety issues and the non-standardization of procedures have limited the clinical application of FMT (6, 11). In contrast, compared with undefined fecal samples, a consortium of harmless, health-associated bacteria may be a viable therapeutic alternative for the treatment of IBD. Bacterial consortium transplantation (BCT) is regarded as being more stable and controlled than FMT (12–14). Using a consortium of 10 bacterial strains that were isolated from fecal samples of healthy mice, we have previously shown that BCT and FMT were comparable in re-establishing mucosal barrier function in mice with intestinal dysbiosis (15). Further studies in rats with trinitrobenzene sulfonic acid (TNBS)-induced colitis showed that this bacterial consortium exhibited anti-inflammatory activity in the intestine of rats and contributed greatly to the reduction of gut permeability, as well as the rapid re-establishment of intestinal microbial equilibrium (16). However, the underlying mechanisms remain unclear.

The intestinal epithelium is built of monolayered columnar epithelial cells that are tightly connected by tight junction proteins such as occludin and claudins. Impaired tight junction protein, which leads to the reduced epithelial barrier integrity, was found in both human IBD and a mouse model with dextran sulfate sodium (DSS)-induced colitis—a well-established model of mucosal inflammation used in IBD studies (17, 18). Although tight junction proteins are normally considered as a part of the physical barrier, they are largely affected by mucosal immune homeostasis within the lamina propria (LP) and the composition of gut microbes and thus can be regarded as a translator between microbiota and immune system (6, 19, 20). Among the immune cells dwelling in intestinal mucosa, γδT cells are rare T-cell subsets that were found to be involved in both pathogenic and protective networks in IBD (21). They have been the targets of recent investigation because of their spontaneous IL-17A expression (22) and interaction with intestinal microbiota (23, 24). γδT cells in intestinal LP were found to be the major source of gut-protective IL-17, and this γδT cell-derived IL-17 promoted the repair of damaged intestinal epithelium through adaptor molecule Act1-mediated regulation of occludin subcellular localization (25). Given the important regulatory role of gut microbes and γδT cells in regulating occludin, we therefore hypothesized that the IL-17A-producing γδT (γδT17) cells might be involved in the protection mechanism of the bacterial consortium on colon mucosa.

In this study, we showed that transplantation of the bacterial consortium resulted in the reduction of gut permeability and improvement of intestinal dysbiosis in mice with DSS-induced colitis; and it also resulted in the elevation of γδT17 cells in colonic lamina propria (cLP) of mice. The IL-17R-Act1-occludin signaling crosstalk was found to be upregulated in mice that received BCT. In addition, specific bacterial groups were found correlated with the expansion or reduction of γδT17 cells. Our results support the possibility that the alliance between gut microbiota and γδT17 cells plays an important regulatory role in IBD, and this study provides new insights for the development of microbiota transplantation therapy.

## Materials and Methods

### Animal Experiments

Male C57BL/6J mice were obtained from the Experimental Animal House of Dalian Medical University, China, where they were maintained under stress-free and specific pathogen-free conditions, under 12 h cycles of light and darkness. Food and water were provided *ad libitum* before experiments. The animal experimental procedures were approved by the Medical Ethics Committee of Dalian Medical University, China (SYXK2015-0002) (16).

To induce acute colitis, 6- to 8-week-old mice were given drinking water containing 3.0% (w/v) DSS (MP Biomedicals) *ad libitum* for 7 days and distilled water for 1 additional day before sacrifice. All the mice were anesthetized before sacrifice. To detect gut permeability, FITC-Dextran (4 kDa, Sigma-Aldrich) were gavaged to mice as previously described (25) 3 h prior to fluorometric analysis of FITC fluorescence in serum.

For BCT, bacterial strains isolated previously from healthy mice were cultured, mixed, and re-suspended in 0.2 ml PBS, according to the method described previously (15). The population of each strain in the mixture is shown in Table S1 in Supplementary Material. The mixture was then gavaged to mouse once a day. Mice of the control and DSS group were gavaged with 0.2 ml PBS as vehicle. The strains used for bacterial transplantation are deposited in the Bacteria Collection of Dalian Medical University, China, and The University’s Institutional Biosafety Committee approved applications to conduct only scientific research with these microorganisms.

### Histopathological Analysis and Measurement of Colon Myeloperoxidase (MPO), Cytokines, and Serum FITC-Dextran

The Disease Activity Index of mice was evaluated according to a previous study (16). After the mice were sacrificed, the colon was extracted, cleaned, weighed, and measured. The colonic tissue samples were preserved in buffered formalin, embedded in paraffin, cut into 5 µm sections, and stained with hematoxylin and eosin (H&E) for histopathological analysis. The rest of the tissue was homogenized in a Greenburger buffer supplemented with protease inhibitors (complete Mini EDTA free; Roche), after sonication for 10 s, the suspension was centrifuged at 8,000 × *g* for 20 min at 4°C. The supernatant was used to quantify the MPO activity (which correlated with the degree of neutrophil infiltration) and the levels of cytokines using ELISA kits (USCN, USA). Peripheral blood of the mice was centrifuged at 1,500 × *g* for 15 min at 4°C to obtain serum. Serum FITC-Dextran was assayed by ELISA (USCN, USA) according to the manufacturer’s instructions (16).

### Immunofluorescence Microscopy

The distal colons of mice were first flushed with PBS, then embedded in Tissue-Tek O.C.T. compound (SAKURA Finetechnical Company) in cryomolds, and snap frozen in liquid nitrogen for cryosectioning. The cryosections were prepared at −20°C with 8 µm thickness on a Leica Cryostat (Leica Microsystems). Sections were mounted on glass slides and fixed in 100% ethanol at 4°C for 30 min followed by 3 min of 20°C acetone fixation at room temperature. After washing in PBS, the slides were blocked in FBS and goat serum for 1 h in room temperature. The tissue sections were stained with a monoclonal occludin antibody sc-5562 (SANTA CRUZ Biotechnology) at 4°C overnight. After washing in PBS, the sections were stained with a goat anti-rabbit IgG Alexa Fluor 488 conjugated secondary antibody (Fcmacs) for 60 min at room temperature. After washing in PBS, the tissue sections were treated with DAPI (Millipore) for 5 min and covered with a coverslip. Fluorescence was observed with a microscope (DM4000B; Leica, Germany) equipped with a digital camera at 40× magnification (25).

### RNA Isolation and Quantitative Real-time PCR (qPCR)

To analyze the mRNA expression of genes, total RNA in mouse colon tissues was extracted by using the RNeasy mini Kit (Qiagen, Hilden, Germany). The complementary DNA (cDNA) was synthesized using the AffinityScript Multiple Temperature cDNA synthesis Kit (Stratagene, La Jolla, CA, USA). Reverse transcription was performed to obtain cDNAs, which were used to detect mRNA expression of *Tjp1, Ocln, il17a*, and *ROR*γ*t* by the specific primers listed in Table S2 in Supplementary Material. The reactions were run on an ABI StepOne Plus Sequence Detection System (Applied Biosystems). The reaction mixture (10 µl) includes 4.5 µl SYBR Premix Ex Taq (Perfect Real Time, TAKARA, Japan), 0.5 µl of forward and reverse primers (30 mM), 2.5 µl of sterile distilled water, and 2.5 µl of cDNA (100 ng/ml). Each sample was run in triplicate, and the mean Ct was determined. The relative mRNA expression was expressed as ΔCt = Ct (target genes) − Ct (calibrator). The expression of the GAPDH gene was used as a calibrator after verification of its stability under current experimental conditions. The relative mRNA expression was calculated as ΔΔCt = ΔCt (test group) − ΔCt (control group) and expressed as fold change (=2^−ΔΔCt^).

### Western Blot (WB)

The total protein was extracted from colon tissues of mice. Equivalent amounts of protein were separated by sodium dodecylsulfate polyacrylamide gel electrophoresis (SDS-PAGE) and were transferred onto nitrocellulose membranes. Antibodies against Occludin, Act1, IL-17RC GADPH (USCN, USA) were used for blotting, and the secondary antibodies conjugated with horseradish peroxidase (HRP, USCN, USA) were used to show the bands. The immune complexes were detected with a WesternBright™ ECL Western Blotting HRP Substrate kit and analyzed with image lab software (Bio-Rad, USA).

### Cell Isolation from cLP and Spleen

The colons of mice were incubated in a 37°C water bath in cell dissociation solution made with HBSS and HEPES (Solarbio) to strip the epithelial cells. Supernatant was discarded, and colonic tissue was then incubated in a digestion cocktail containing HBSS, FCS (10%, Gibco), type IV1 collagenase (1 mg/ml), DNaseI (0.5 mg/ml), and dispase (0.5 mg/ml, all from Sigma-Aldrich) in a 37°C water bath. After that, the digested tissue was processed through a 70 mm filter (Falcon) and washed before lymphocytes were separated by the methods used previously (20, 24). γδT cells were purified from the spleens of mice using the Mouse TCRγδ^+^ T-cell Isolation Kit and the miniMACSTM (Miltenyi Biotec, Germany).

### Cell Culture

Cells were put into single-cell suspensions at 1–2 × 10^6^ cells/ml and were plated for a final volume of 200 µl and re-stimulated with media alone or with media and bacterial components. A total of 1 ml of the bacterial consortium, or culture broth of *Bifidobacterium longum* ATCC 15707 and *Bacillus cereus* ATCC 14579, was sonicated to release the cellular components, after that, the mixture was centrifuged at 8,000 rpm for 2 min, and different volumes of the supernatant were added to the single-cell suspension after filtration by a 0.2 μm-filter. After co-culture for 20 h, the IL-17A level in the cell culture supernatant was detected by ELISA (USCN, USA).

### Flow Cytometry

For IL-17A staining, cells were first stimulated with 2 µl/ml cell stimulation cocktail (500, plus protein transport inhibitors; eBiosceince) in the presence of 100 µl Intracellular Fixation and Permeabilization Buffer Set (eBioscience) Golgi-plug (eBioscience) for 12 h in complete medium. Surface staining was then performed in the presence of Fc-blocking antibodies (albumin, bovine, fraction V-Coolaber) and using αCD4 (anti-mouse CD4 FITC, 11-0041; eBioscience), αCD3 (anti-mouse CD3 FITC, 11-0032-80; eBioscience), α-γδTCR (anti-mouse gamma delta TCR PE, 12-5711; eBioscience), and α-TLR2 (anti-mouse CD282 eFluor^®^660; 50-9021, eBioscience). Cells were then fixed and permeabilized with the Cytofix/Cytoperm kit (BD) followed by intracellular staining using antibodies against IL-17A (anti-mouse/rat IL-17A APC, 17-7177; eBioscience). All samples were collected (Accuri C6; BD Bioscience, USA), and data were analyzed with Flow Plus 1.0.264.15 Software.

### DNA Isolation from Colonic Contents

The metagenomic DNA in the colon content of mice was isolated by the QIAamp DNA stool mini kit (Qiagen, Germany), and a NanoDrop 2000 spectrophotometer was used to measure the purity and concentration of the DNA (Thermo, USA) (16).

### Library Construction, Sequencing, and Data Analysis

The V3–V4 region of 16S rDNA was amplified with universal primers. The PCR products were then quantified by electrophoresis on a 1.5% agarose gel and purified with the QIAquick Gel Extraction kit (Qiagen). Sequencing and data analysis were subsequently performed on an Illumina HiSeq platform by Novogene (Beijing, China) using a method described previously (26). Briefly, we used Ribosomal Database Project Classifier 2.8 to perform the assignment of all sequences at 50% confidence after the raw sequences were identified by their unique barcodes. OTUs present in 50% or more of the colon content samples were identified as core OTUs. PLS-DA of core OTUs was performed using Simca-P version 12 (Umetrics), and a heatmap was generated using Multi-Experiment Viewer software to visualize and cluster the bacterial community into different groups. Community diversity was measured by the Shannon–Weiner biodiversity index (Shannon index).

### Statistics

All data were evaluated as mean ± SEM (*n* = 5). Statistical analysis of the quantitative multiple group comparisons was performed using one-way analysis of variance (and non-parametric), followed by Tukey’s test (compare all pairs of columns); when two groups were compared, the non-parametric *t*-test was performed with the assistance of GraphPad Prism 5 (Graph Pad Software, La Jolla, CA, USA). Results were considered to be statistically significant with *p* < 0.05.

## Results

### The Bacterial Consortium Protected against DSS-Induced Colitis in Mice

**Figure 1:**
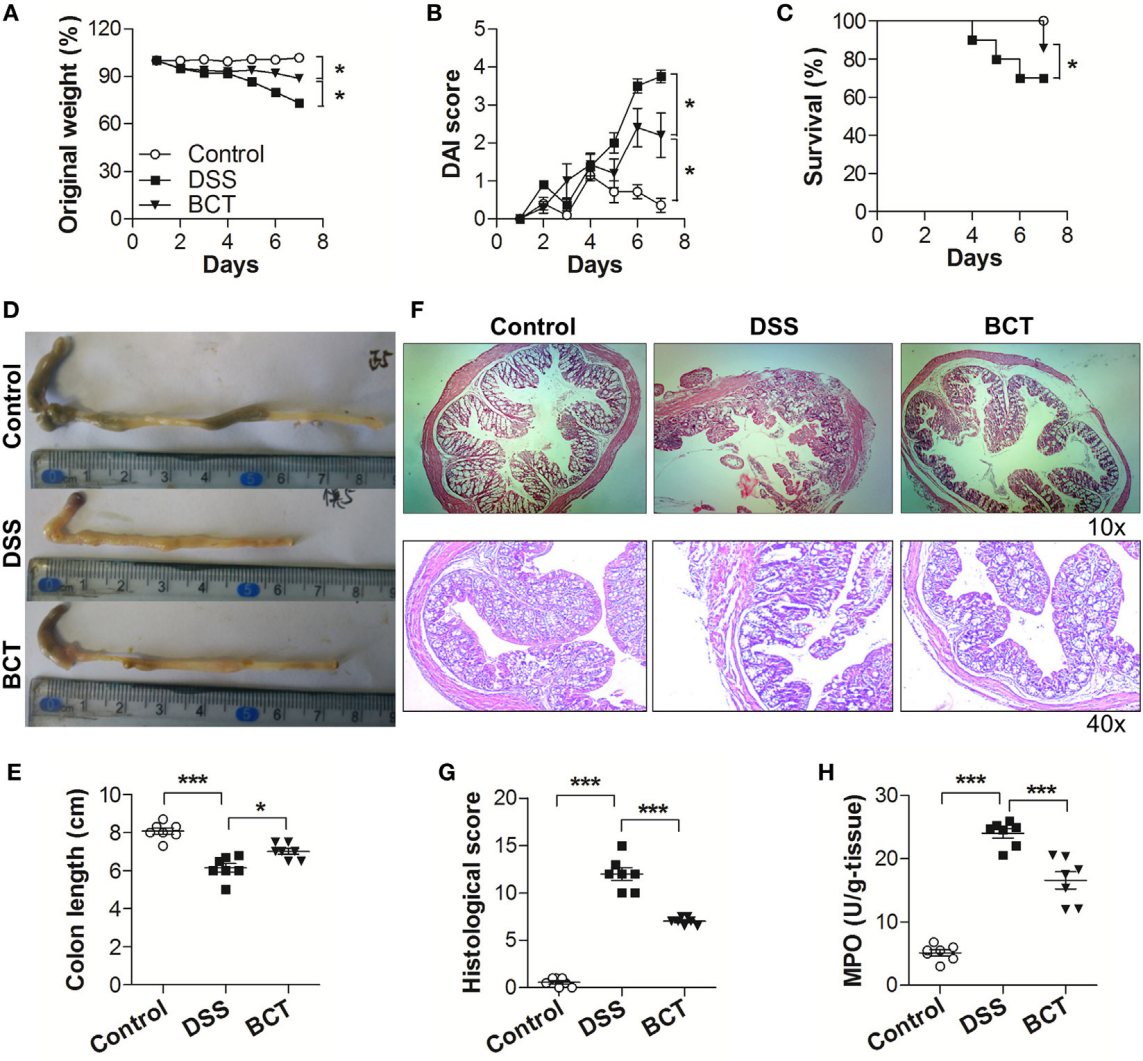
BCT ameliorated DSS-induced colitis in mice. The effect of BCT on DSS-treated mice was assessed by **(A)** change of original weight; **(B)** Disease Activity Index score; **(C)** survival rate; **(D,E)** the colon length was determined after sacrifice; **(F)** H&E-stained results for the sections of mouse colon; **(G)** histopathological analysis of the H&E-stained sections; **(H)** the myeloperoxidase of colonic tissue was detected by ELISA following the manufacturer’s protocol. All data were evaluated as mean ± SEM (*n* = 5). Statistical analysis of the quantitative multiple group comparisons was performed using the one-way analysis of variance followed by Tukey’s test, *p < 0.05, ***p < 0.001.

We first investigated the potential therapeutic action of the bacterial consortium in mice with DSS-induced colitis, although it had already shown protective effects in TNBS rat models (16). Mice subjected to 3.0% DSS developed severe illness characterized by profound and sustained weight loss (Figure 1A), bloody diarrhea, and wasting syndrome resulting in the increase of the disease index and mortality (Figures 1B,C). Transplantation of the bacterial consortium ameliorated the weight loss and disease severity, with a survival rate of 85.7%, which was significantly higher than the survival rate of DSS-treated mice (70.0%) (Figures 1A–C). The average colon length of the consortium-treated mice was longer than that of the DSS group (Figures 1D,E) with edema-induced shortening. Histological evaluation of the distal colon of the consortium-treated group showed no signs of macroscopic and transmural inflammation (Figures 1F,G); in contrast, the colon of DSS-treated mice showed significant transmural inflammation involving all layers of the colonic wall and marked increase in the thickness of the muscular layer. The neutrophil infiltration, which was correlated with a significant increased colonic MPO level, was detected in DSS-treated mice but was much mitigated in the bacterial consortium-treated mice (Figure 1H).

### BCT Decreased the Gut Permeability of DSS-Treated Mice by Upregulating the Expression of Tight Junction Protein Occludin

**Figure 2:**
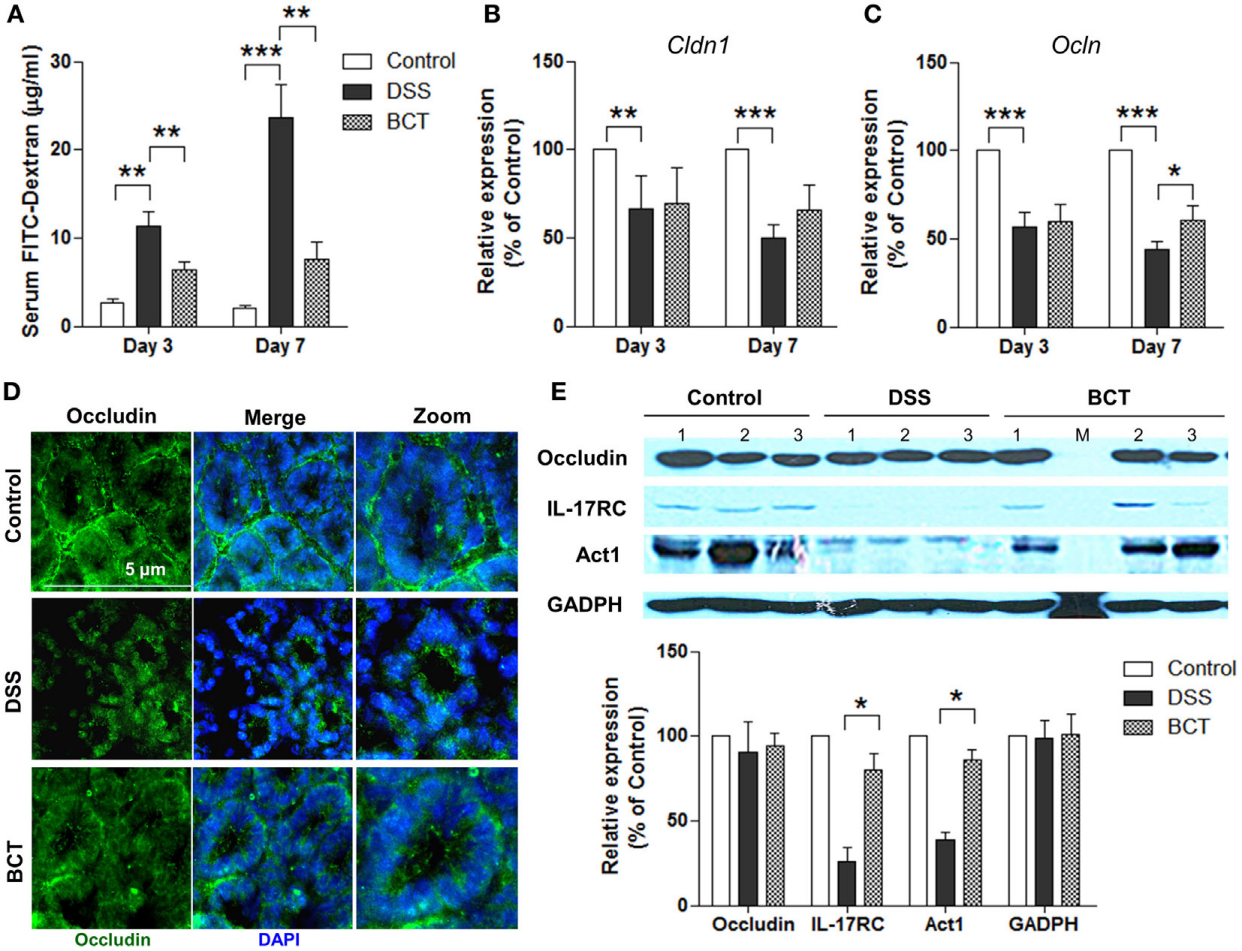
BCT enhanced intestinal barrier function of colitis mice by regulating IL-17-Act1-occludin axis. **(A)** Detection of FITC-dextran in serum of mice by ELISA. **(B,C)** The relative expressions of genes encoding claudin and occludin proteins in colon tissues of mice detected by qPCR. **(D)** Representative immunofluorescence images of occluding (green) and DNA (blue) of distal colon segments from mice of different groups. The third column represents a magnified image. **(E)** The expression of occludin, IL-17RC, and Act1 detected by Western blot with GADPH as internal control. All values are mean ± SEM (*n* = 5). Statistical analysis of the quantitative multiple group comparisons was performed using the one-way analysis of variance followed by Tukey’s test, *p < 0.05, **p < 0.01, ***p < 0.001.

To further characterize the protective effects of the bacterial consortium on the epithelial layer in DSS-treated mice, we quantified the permeability by orally administering FITC-dextran to mice and measuring the serum levels. Results showed that the diffusion of FITC-dextran through the epithelium after DSS treatment was significantly increased in the intestines of mice (Figure 2A) on day 3 and day 7, and it was compromised in the BCT group of mice. The tight junction complex claudins and occludins play crucial roles in regulating gut permeability and the epithelial paracellular pathway (27). We therefore tested the transcription levels of *Cldn1* and *Ocln* in mouse colon tissues by quantitative real-time PCR (qPCR). DSS treatment resulted in a decrease in mRNA expression level of tight junction complex (Figures 2B,C), and it was enhanced in the bacterial consortium-administrated mice. Although the transcripts of *Ocln* mRNA in BCT mice were still lower than in control mice when tested by immunofluorescence, we found that the cellular localization of occludin was less impacted by DSS treatment, which was localized on the apical surface of the cell (Figure 2D), in contrast to the intracellular occludin staining observed in the DSS group of mice. Previous study showed that cellular localization of occludin could be regulated by the IL-17R-Act1 signaling pathway. We therefore tested the expression of IL-17RC and Act1 in colon tissue by WB and found that BCT improved the expression of these proteins, which was significantly decreased in DSS-treated mice (Figure 2E).

### The Intestinal γδT17 Cells Were Upregulated by BCT

**Figure 3:**
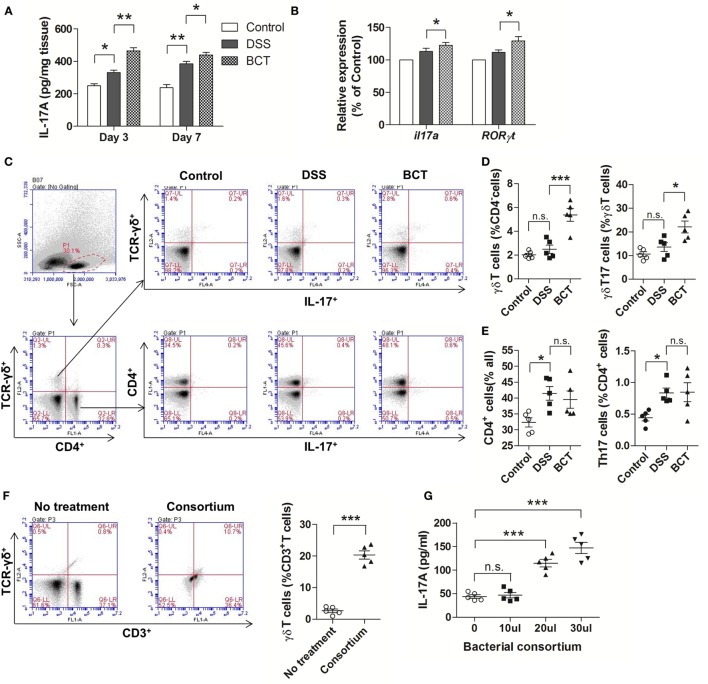
BCT upregulated IL-17^+^ γδT cells in colonic lamina propria (cLP) of colitis mice. **(A)** The IL-17A concentration in each animal group was detected in supernatants of colonic tissue homogenates by ELISA. **(B)** The relative mRNA expression of *il17a* and *ROR*γ*t* in colon tissues of mice detected by qPCR. **(C)** Representative flow cytometry plots of CD4^+^ T cells identified using side scatter and forward scatter plots (left panel) and TCR-γδ^+^ expression. And the representative flow cytometry plots of γδT17 cells (TCR-γδ^+^ IL-17A^+^) (upper panel) and Th17 cells (CD4^+^IL-17A^+^) (bottom panel). **(D)** Percentage of γδT cells and γδT17 cells in γδT cells from cLP of different mice. **(E)** Percentage of CD4^+^ T cells and the percentage of Th17 cells in cLP of mice from different groups. **(F)** Representative flow cytometry plots and evaluation of the percentage of γδT cells among cLP cells treated or un-treated with 30 µl of the bacterial consortium *in vitro*. **(G)** IL-17A concentration in supernatant from cLP cells treated or un-treated with different volumes of the bacterial consortium *in vitro* detected by ELISA. All values are mean ± SEM (*n* = 5). Statistical analysis of the quantitative multiple group comparisons was performed using the one-way analysis of variance followed by Tukey’s test, *p < 0.05, ***p < 0.001, n.s., not significant.

Previous study had shown that after acute intestinal injury, the intestinal-protective IL-17A could regulate the tight junction protein occludin to limit excessive permeability and maintain barrier integrity (25). We therefore tested the IL-17A levels in the colon mucosa by ELISA. Our results showed that DSS-caused injury induced an elevation of IL-17A in colon tissue, and this response could be detected as early as day 3 (Figure 3A). We detected an even higher concentration of IL-17A in mice that received BCT at both day 3 and day 7. In addition, the mRNA expressions of *il17a* and RORγt were significantly upregulated in BCT mice compared with that of the DSS group (Figure 3B), which confirmed the elevation of IL-17A secretion in colon mucosa. Studies have shown that γδT cells in colon mucosa are the primary producers of early protective IL-17A and, thus, play important roles in maintaining epithelial barriers. To investigate whether the protection effects of the bacterial consortium were associated with the upregulation of γδT17 cells, we analyzed the major intestinal IL-17-producing T-cell population in different mouse groups at day 3. Increase in γδT (CD4^−^TCR-γδ^+^) cells, as well as γδT17 (CD4^−^TCR-γδ^+^ IL-17A^+^) cells, was detected in BCT mice (Figures 3C,D). Further investigation revealed that the promoting effect of the bacterial consortium was only on γδT17 cells but not on the CD4^+^ cells or Th17 (CD4^+^ IL-17A^+^) cells (Figures 3C,E). We further isolated the total cLP cells and tested the effects of the bacterial consortium on the expansion of γδT cells and found that the population of γδT cells increased dramatically (*p* < 0.0001) upon treatment with bacterial products derived from the consortium (Figure 3F). An elevated IL-17A concentration was detected in the cell culture supernatant that was treated with the bacterial consortium when measured by ELISA (Figure 3G).

### The Bacterial Consortium Promoted Expansion of TLR2^+^ γδT Cells

**Figure 4:**
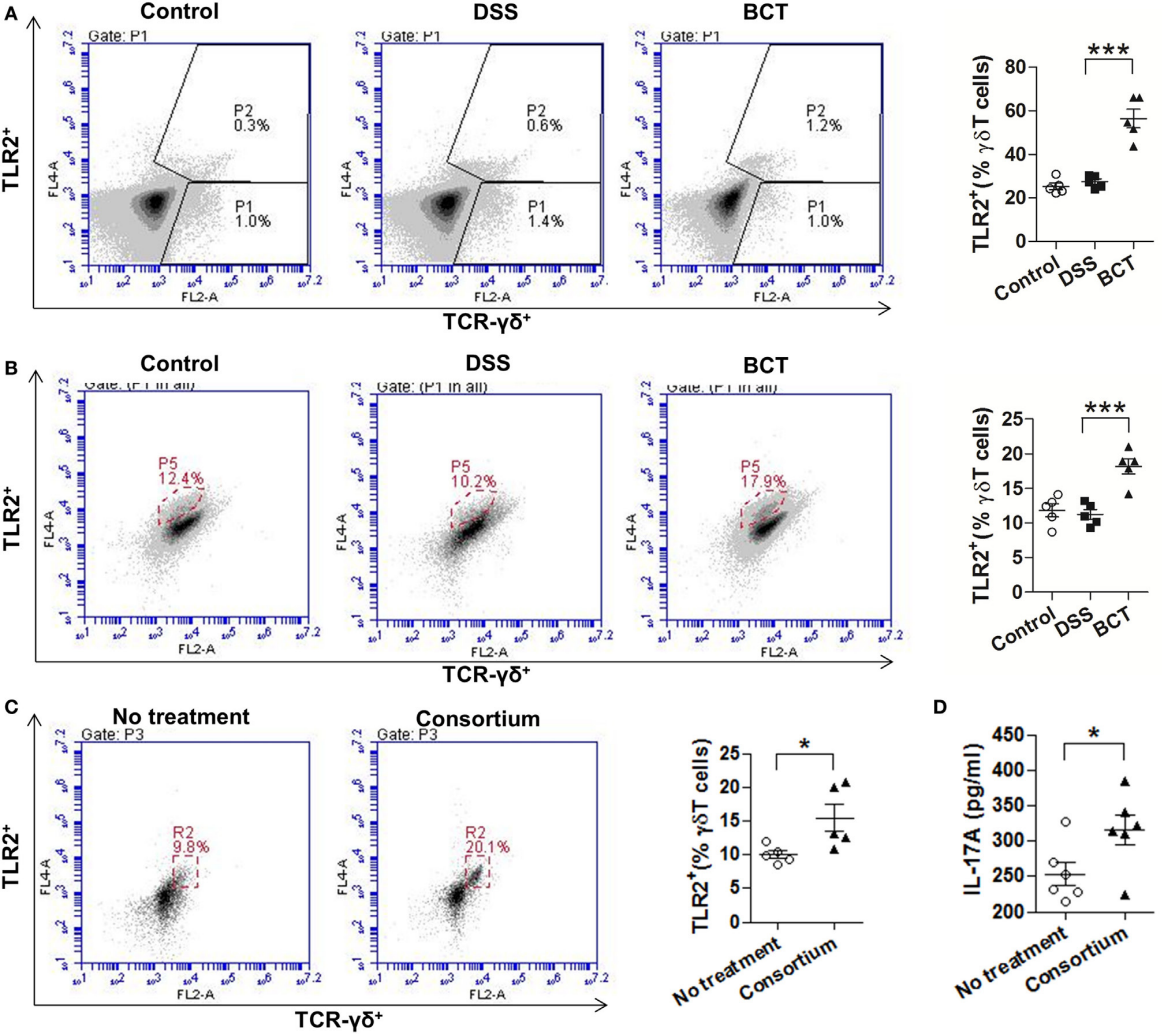
The bacterial consortium promoted the expansion of TLR2^+^ γδT cells. **(A)** TLR2^+^ γδT cells in colonic lamina propria (cLP) of colitis mice were upregulated post-transplantation of the bacterial consortium. Left panel, representative flow cytometry analysis of TLR2^+^ γδT (TLR2^+^TCR-γδ^+^) cells in cLP of mice from different groups at day 7. Right panel, the proportion of TLR2^+^ γδT cells in total cLP γδT cells. **(B)** TLR2^+^ γδT cells in spleen of colitis mice were upregulated post-transplantation of the bacterial consortium. Left panel, representative flow cytometry analysis of MACS-purified splenic γδT cells from mice of different groups at day 7; right panel, the proportion of TLR2^+^ γδT cells in total splenic γδT cells. **(C)** Effects of the bacterial consortium on TLR2 expression of purified splenic γδT cells. Representative flow cytometry plots and evaluation of the percentage of TLR2^+^ γδT cells among γδT cells treated or un-treated with 30 µl of the bacterial consortium *in vitro*. The γδT cells were purified by MACS from the spleen of C57BL/6J mice at day 7, which belonged to the control group. **(D)** IL-17A concentration in the supernatant of purified γδT cells treated or un-treated with 30 µl of the bacterial consortium *in vitro* detected by ELISA. All values are mean ± SEM (*n* = 5). Statistical analysis of the quantitative multiple group comparisons was performed using one-way analysis of variance followed by Tukey’s test; when two groups were compared, the non-parametric *t*-test was performed by the assistant of GraphPad Prism 5. *p < 0.05, **p < 0.01,***p < 0.001.

Previous study (28) had shown that γδT17 cells express toll-like receptors TLR2, which selectively expands in response to bacterial products. We therefore examined the expression of TLR2 by cLP single-cell suspension with or without treatment with the bacterial consortium. Results showed that, upon treatment of the bacterial consortium (Figure 4A), the TLR2^+^ γδT cells were un-regulated (Figure 4A). We further purified the splenic γδT cells of mice from different groups by MACS and found that BCT significantly increased the expression of TLR2 by γδT cells (Figure 4B). When purified γδT cells were challenged with the bacterial consortium *in vitro*, we detected an expansion of TLR2^+^ γδT cells (Figure 4C), as well as an elevated IL-17A concentration in the cell culture supernatant (Figure 4D), which suggests that the TLR2 signaling pathway may be involved in the secretion of IL-17A from γδT cells.

### BCT Benefited the Re-Establishment of Intestinal Bacterial Equilibrium

**Figure 5:**
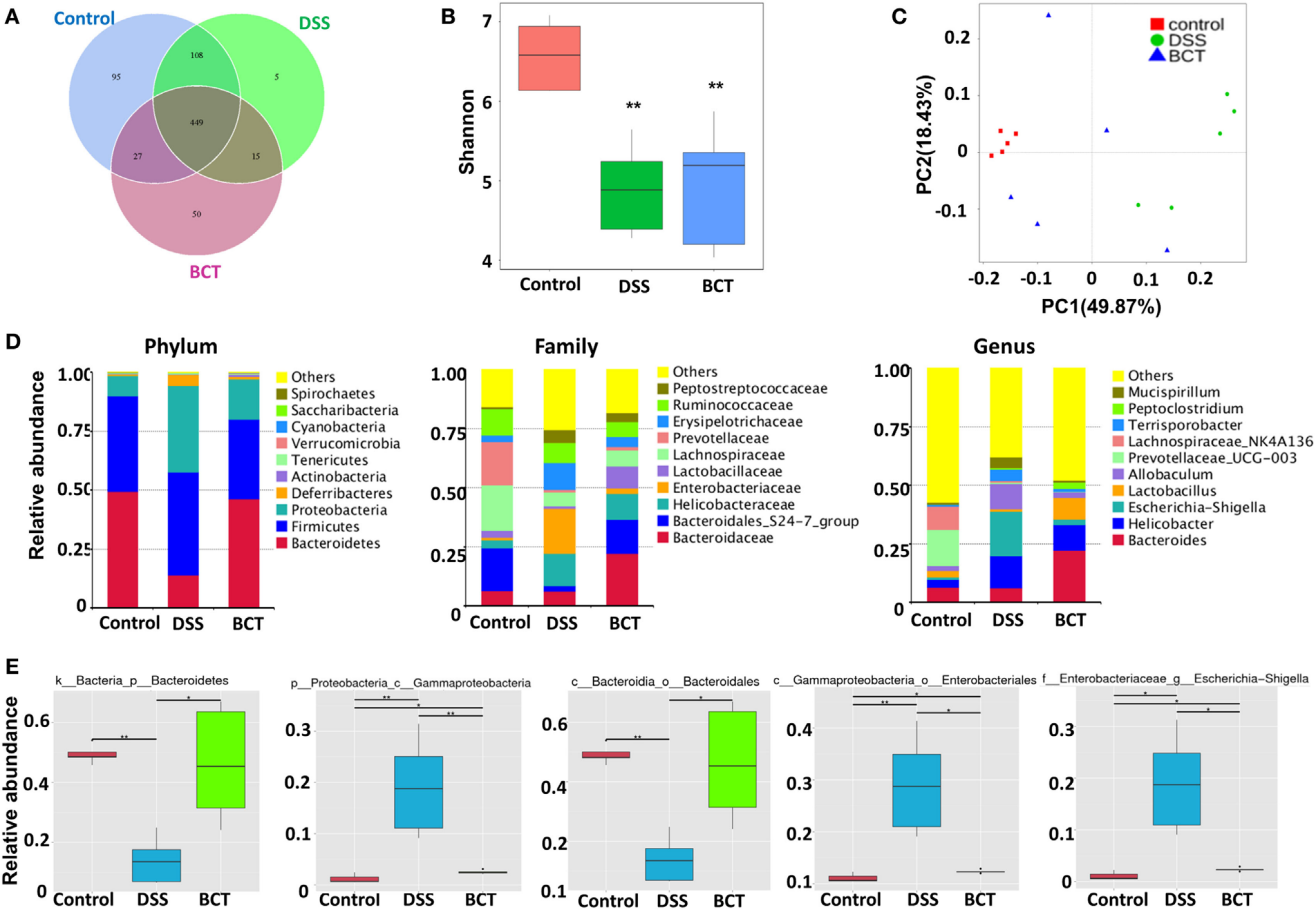
BCT modulated intestinal bacterial composition in model mice. **(A)** Venn diagram of shared and independent bacterial OTUs in different experimental groups (*n* = 5). **(B)** Comparison of the Shannon index of different groups. **(C)** Principal Coordinate Analysis based on weighted Unifrac distances among different samples. PC1 and PC2 account for 68.30% of the variation. **(D)** The composition of bacterial composition in different experimental groups at Phylum, Family, and Genus levels. **(E)** The specific bacterial groups that are significantly manipulated by BCT. The MetaStat method was used to identify the significantly reduced or elevated microbial groups by DSS treatment and was reversed by BCT. k, kingdom; p, phylum; c, class; o, order; f, family; g, genus; All values are mean ± SEM (*n* = 5). *adjusted *p* value <0.05; **adjusted *p* value <0.01.

We have previously shown that administration of the bacterial consortium promoted the recovery of intestinal microbial equilibrium in mice with ceftriaxione-induced dysbiosis, as well as in rats with TNBS-induced colitis (15, 16). However, these results were based on PCR-DGGE technique, which gave a low resolution of intestinal microbiota. Here, we further adopted metagenomic analysis of the V3–V4 region of 16S rRNA gene sequences to systematically characterize the composition of the intestinal microbiota in mouse intestine post-BCT. Results showed that the overall OTUs of intestinal bacteria differ between groups (Figure 5A). Compared with the control group, the DSS-treated mice and BCT mice had a decreased number of OTUs. The three groups share the same 449 OTUs, but there are significant differences between every two groups (Figure 5B), with the most significant difference observed between the control group and the DSS group. Transplantation of the bacterial consortium resulted in obvious modification of the bacterial structure of mouse intestine as confirmed by the Principal Coordinate Analysis (Figure 5C). Changes were observed not only on the phylum level but also on the levels of order, class, family, and genus (Figure 5D; Figures S1 and S2 in Supplementary Material). A significant decrease in the abundance of *Bacteroidetes* (phylum) and *Bacteroidales* (order) and an elevated abundance of *Gammaproteobacteria* (class), *Enterobacteriales* (order), *Escherichia–Shigella* (genus), etc., were detected in DSS-treated mice (Figure 5E; Figure S3 in Supplementary Material). In contrast, transplantation of the bacterial consortium resulted in the correction of these bacterial groups, especially the genus of *Escherichia–Shigella*, which may contribute to the re-establishment of intestinal equilibrium.

### Biomarkers in Each Group

**Figure 6:**
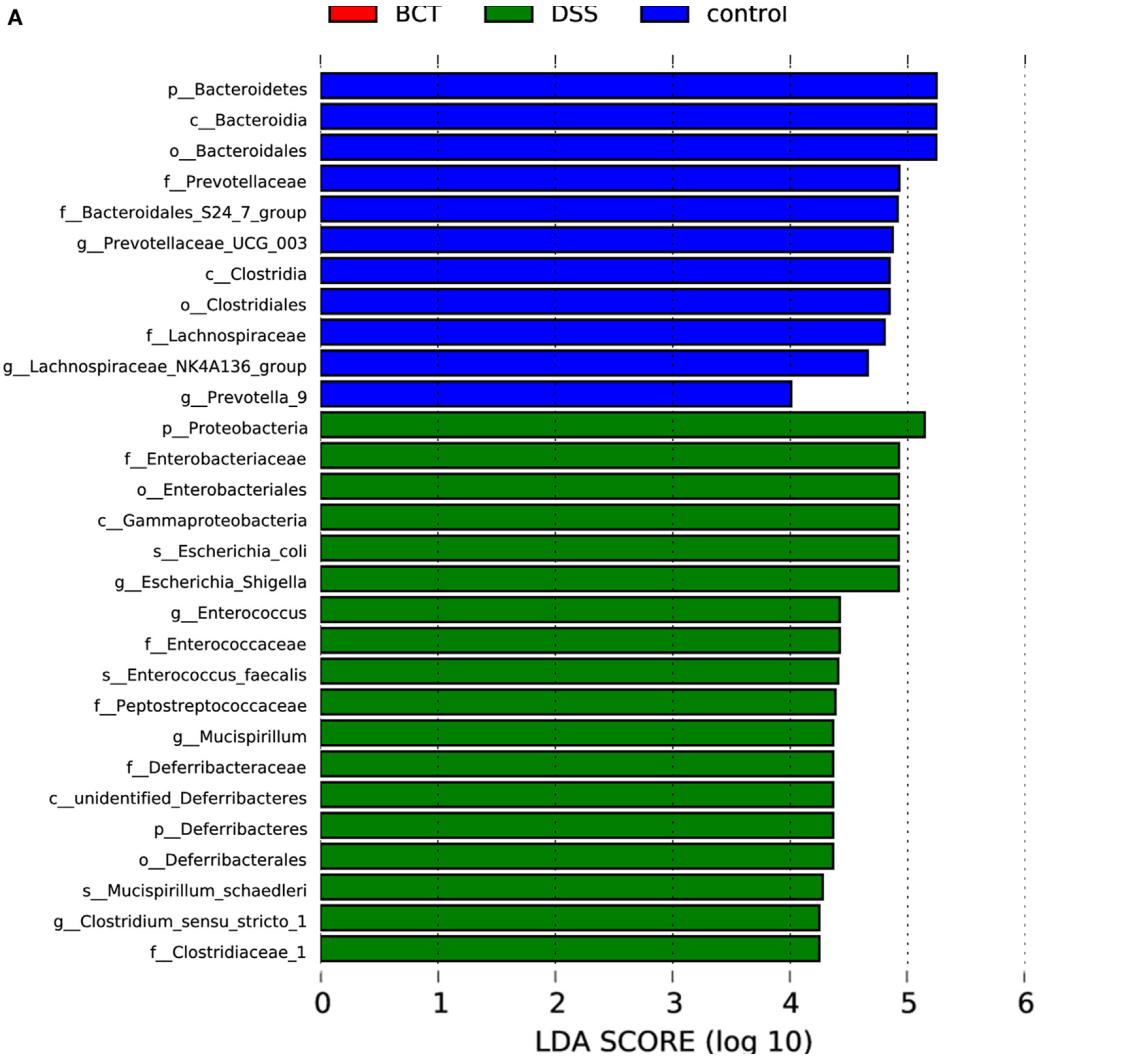

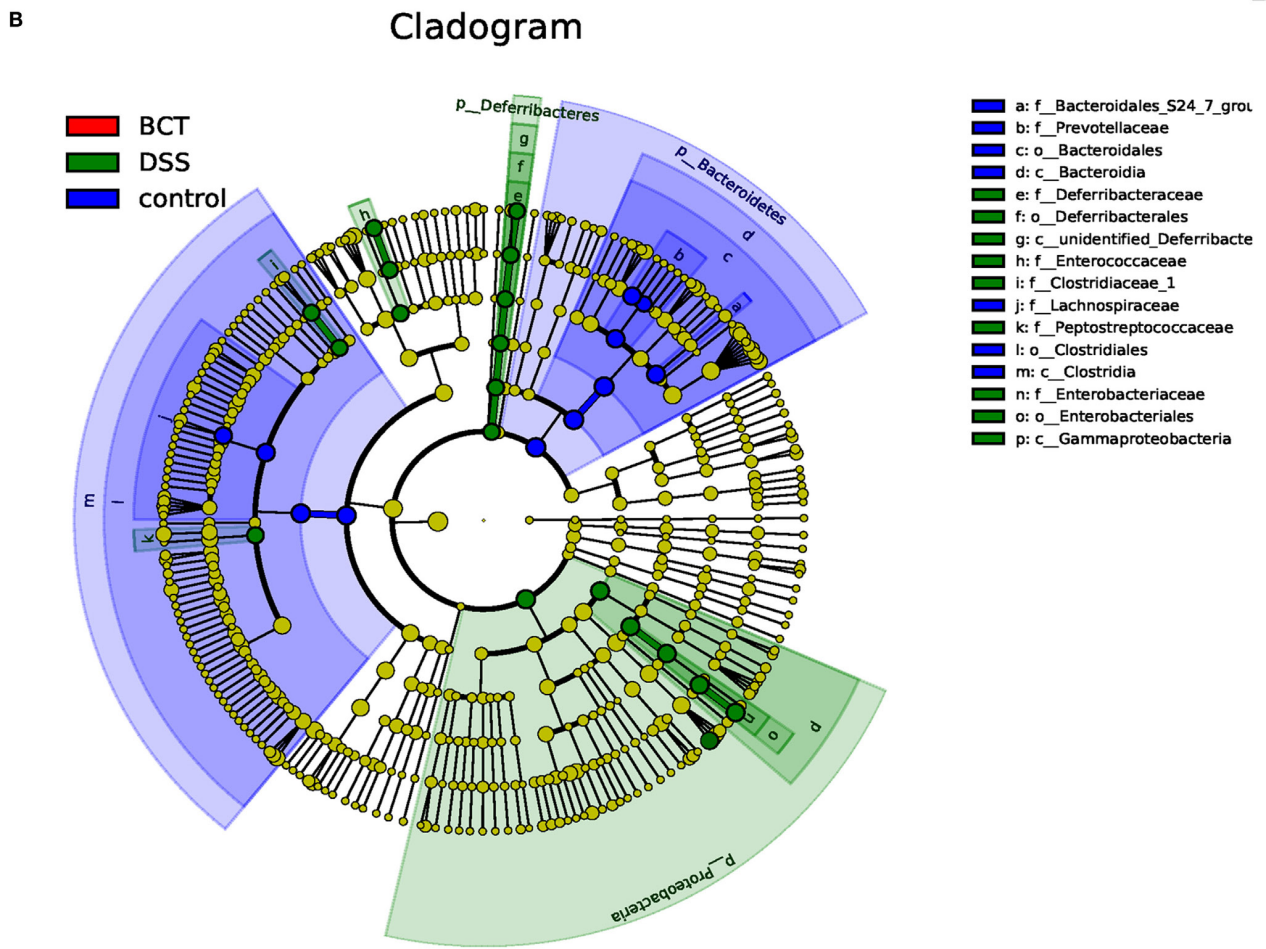
LEfSe analysis of intestinal microbiota among different mice groups. **(A)** LEfSe identified the most differentially abundant bacterial taxons among groups. Group-specific enriched taxa are indicated with a positive LDA score bar with different colors. Only taxa meeting an LDA significant threshold >4 are shown. **(B)** Taxonomic cladogram obtained from LEfSe analysis of 16S rDNA sequences. The brightness of each dot is proportional to its effect size.

The metagenome analysis LEfSe approach was applied to identify the key phylotypes responsible for the differences between groups. *Bacteroidetes* and *Clostridia*, which were most abundant in the control mice, and *Proteobacteria, Diferribacteres*, and *Enterobactriales*, which were most abundant in the DSS mice, were the dominant phylotypes that contributed to the differences between the intestinal microbiota of control and DSS-treated mice (Figure 6). We did not detect any biomarkers in BCT mice, which suggest that the manipulation of microbiota by the bacterial consortium mainly may contribute to the recovery of disrupted intestinal homeostasis, rather than establishing a new unique microbiota.

### Changing of Specific Bacterial Groups Was Correlated with γδT Cells

**Figure 7:**
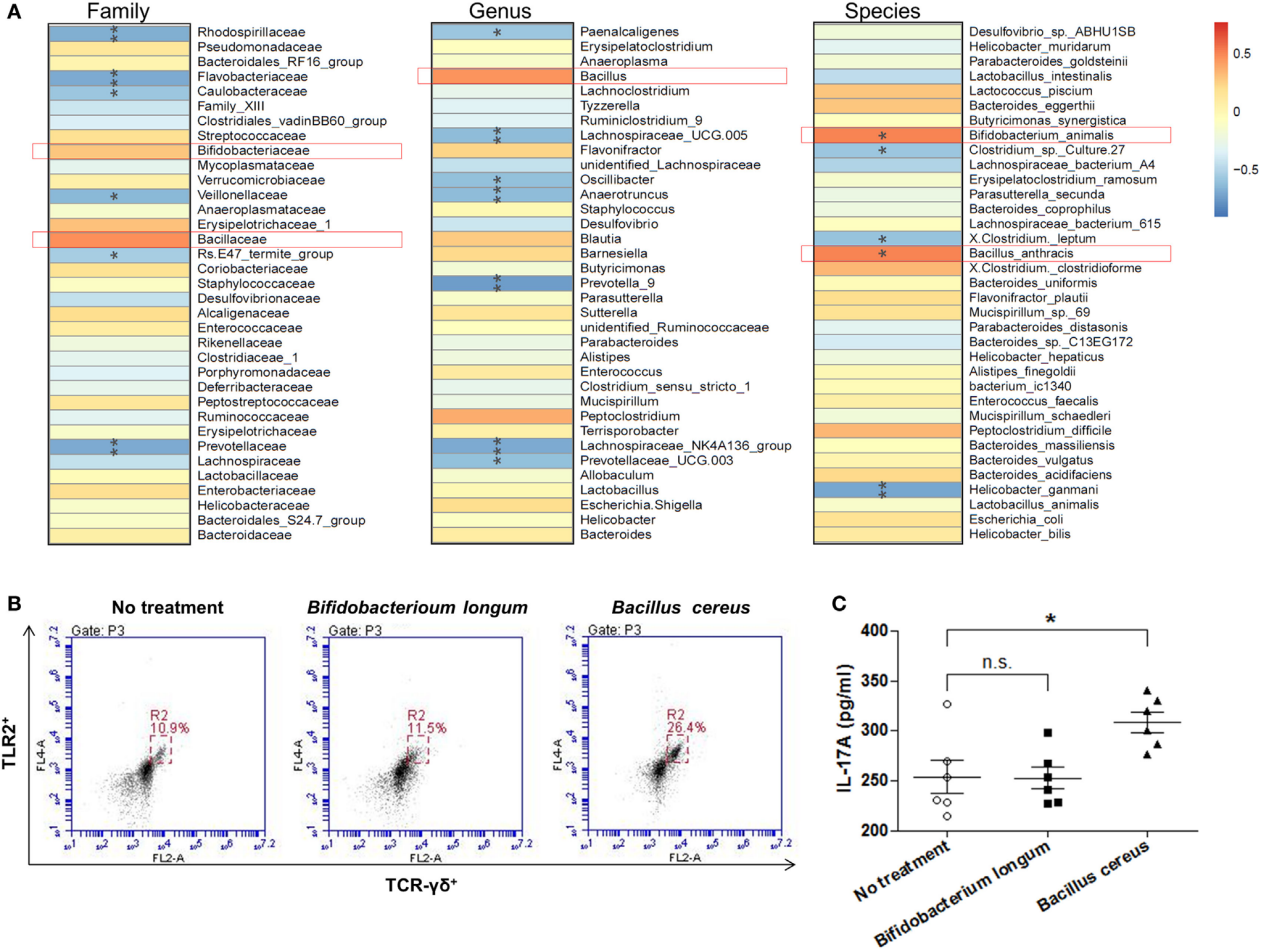
The correlation between intestinal bacterial groups and γδT cells. **(A)** The correlation between the relative abundance of different microbial groups at family, genus, and species level and the population of γδT17 cells was tested by Spearman correlation method. The positive correlation was displayed as correlation value >0, and the negative correlation was displayed as correlation value <0; statistically significant correlation was displayed as *p < 0.05; **p < 0.01. **(B)** Flow cytometry analysis of TLR2 expressing γδT cells among purified splenic γδT cells treated or un-treated with specific bacterial strain. **(C)** IL-17A concentration in supernatant from purified splenic γδT cells treated or un-treated with specific bacterial strain *in vitro* detected by ELISA. All values are mean ± SEM (*n* = 5). The non-parametric *t*-test was performed between two groups by the assistant of GraphPad Prism 5. *p < 0.05, n.s., not significant.

With the aim of clarifying how changing of intestinal microbial structure affects γδT cells, we analyzed the spearman correlation between intestinal bacterial groups and γδT17 cells. It was found that bacteria belonging to the families of *Rhodospirillaceae, Flavobacteriaceae, Prevotellaceae*, etc., were negatively correlated with the upregulation of γδT17 cells in mouse intestines (Figure 7A), especially the genus of *Lachnospiraceae* UCG.005, *Lachnospiraceae* NK4A136, *Prevotella* 9, *Prevotella* UCG.003, and the species of *Helicobacter ganmani*. In contrast, bacterial groups, such as the families of *Bifidobacteriaceae* and *Bacillaceae*, were positively correlated with γδT17 cells, especially the species of *Bifidobacterium animalis* and *Bacillus anthracis*, the correlation between these two bacterial species and γδT17 cells are statistically significant (*p* < 0.05). We further tested the effects of culture broth of single bacterial strains, including *Bifidobacterium longum* and *Bacillus cereus*, on purified splenic γδT cells. Interestingly, promotion of TLR2 expression and secretion of IL-17A by γδT cells were observed when the *Bacillus cereus* strain was treated, however, the *Bifidobacterium longum* strain did not show similar effects on γδT cells (Figures 7B,C).

## Discussion

The sustained immune responses caused by intestinal dysbiosis contribute significantly to the pathogenesis and development of IBD. Manipulation of the disturbed microbiota has therefore become a promising therapeutic means for IBD prevention and treatment. In recent years, FMT had been applied clinically in the US, China, and other countries; however, the outcomes were inconsistent (29). In addition, the potential risks of non-conditional pathogens in feces of donors and the attitude of patients toward FMT obstructed its development. As a new generation of bacterial therapy, BCT also has the potential for targeted restoration of the intestinal ecosystem, and the safety and manageability of this therapy suggested a significant step toward precision medicine (30). To this end, research has been carried out to test whether defined microbiota is protective in animal models infected by *C. difficile* (31) or *Salmonella* (32), and the results are promising. Our previous work also showed that a simple, defined microbial consortium can promote the recovery of intestinal microbial equilibrium in mice with ceftriaxone-induced dysbiosis (15). Transplantation of this bacterial consortium also showed effective anti-inflammatory activity on TNBS-induced colitis and upregulation of intestinal barrier function in rats (16), but the specific mechanism remains unknown.

In this study, we further analyzed the BCT effects on intestinal barrier function of DSS-treated mice and found that the tight junction protein occludin was specifically affected. Changing of the expression and the intracellular localization of occludin may contribute mainly to the improvement of the intestinal barrier function. This result suggests an emerging mechanism that the changing of intestinal microbial structure by BCT may affect the occludin regulation system. Intestinal T lymphocytes play a critical role in the mucosal immune system regulation by providing immune surveillance of the epithelium. Among them, the tissue-resident γδT cells are very important members that are considered as both protective and pathogenic T cells in IBD. The protective γδT cells were found to accumulate during DSS or pathogen-induced intestinal inflammation and produce KGF and IL-22, which promotes tissue repair and epithelial cell healing (33). The pathogenic role of γδT cells is mainly due to their production of IL-17, which can induce Th17 differentiation through the inflammatory DC-mediated production of IL-6 and IL-23. And, the Th17 cell-derived IL-17, IL-21, and IFN-γ promote MMP and NO production to induce inflammation and tissue damage (34, 35). However, a recent study demonstrated that IL-17A derived by γδT cells in the cLP promoted epithelial barrier function during DSS-mediated injury and protected the mice from excessive gut permeability (25). Given the previously suggested role of IL-17A in maintaining barrier function of epithelial tissues and the fact that the clinical trials targeting IL-17A were ineffective in treating CD (36, 37), this new finding therefore proves that IL-17A produced by γδT cells is protective, and its effects of maintaining barrier integrity may be exerted mainly through an IL-17R-ACT1-occludin pathway in epithelial cells. Our investigation into this pathway confirmed that the regulation of occludin and the enhancement of barrier function of colon mucosa after BCT are correlated with the expression changes of IL-17A, IL-17RC, and ACT1. And, to test whether γδT cells contributes to the higher levels of IL-17A in colonic tissue, we further tested the T-lymphocyte subpopulations by flow cytometry. The results confirmed that γδT cells but not the Th17 cells are the major source of IL-17A. DSS induced a compensatory accumulation of γδT17 cells in cLP, and this proportion was upregulated after transplantation of the bacterial consortium. The promotion of IL-17A producing γδT cells, therefore, highlighted the regulation mechanism of BCT on intestinal barrier function.

However, it is still unknown how BCT promoted IL-17A production from γδT cells. When tested *in vitro*, we found that the BCT-promoted IL-17A production from γδT cells was closely associated with the upregulation of TLR2 expression. This is in line with prior reports (38, 39) that the TLR2 pathway is critical for recognizing microbial products and activating the innate immune system in response to altered microbiota. Therefore, changing of the intestinal microbial structure may contribute to the mucosal immune response of γδT cells. This idea can be further supported by recent studies that commensal microbiota affects ischemic stroke outcome by regulating intestinal γδT cells (23). Gut commensal microbes were also found essential in maintaining the homeostasis of liver-resident γδT17 cells (24). We therefore tested the intestinal microbial structure of mice from different experimental groups by 16S rDNA pyrosequencing. A dramatically dispersed intestinal microbiota in DSS-treated mice was detected, and this skewed homeostasis was corrected through transplantation of the bacterial consortium.

The deleterious roles of certain bacteria toward the intestinal damage and development of IBD have been proposed. For example, the clade of *Enterobacteriaceae*, particularly *Escherichia*/*Shigella*, has been found to be significantly increased in IBD patients and closely associated with intestinal inflammation (40). The genera *Escherichia*/*Shigella* were also found to be highly enriched in ileal CD patients above the general abundance of CD patients (41). Genera of *Mucispirillum*, which are increased during inflammation, have been suggested to be mucus-dwelling commensals that can cause disease, so called-pathobionts, because under some conditions, the immune system mounts an IgG response against them (42). Significant association of *Prevotella* with CD has been demonstrated by Said et al. (43), and their further studies supported the possibility that the increase of *Prevotella* contributes, at least partially, to the genetic susceptibility to CD (44). In addition, *Helicobacter ganmani*, which are some of the most prevalent bacterial contaminants of laboratory mice, were found associated with alterations in inflammatory cytokines in IL10-deficient mice (45). In accordance with these studies, we demonstrated that the abundance of these bacterial groups was suppressed in the gut of model mice received BCT. Importantly, we observed a significantly negative correlation between *Prevotella* spp. and *Helicobacter ganmani* with γδT17 cells. Interestingly, we also detected a negative correlation between certain *Lachnospiraceae* groups (UCG.005 and NK4A136) with γδT17 cells. The extensive diversity of this family (46) give them divergent resilience to colitis events (42), because promotion of *Lachnospiraceae* strains has been shown to restrict intestinal inflammation (47). Our results thus suggest a previously unidentified physiological difference in this group that may be responsible for the development of colitis.

In contrast, BCT promoted some bacterial groups, which were significantly depleted in DSS-treated mice. And, these groups have been implicated to be beneficial microbes in the gut, which may have evolved mechanisms to ameliorate intestinal inflammation and experimental colitis (48, 49). For example, IBD patients were found to have a decreased population of *Lactobacillus* compared to healthy controls. Many studies support that the colonization of *Lactobacillus* strains accounts for the resistance to DSS-induced colitis of animal models (50). Analogously, in our study, we found a decreased proportion of *Lactobacillus* in DSS-treated mice, while upon BCT, the *Lactobacillus* population was obviously increased (Figure 5D). Species of *Bifidobacterium* are also well-known as beneficial microbes in combating intestinal inflammation. Particularly low numbers and diversity of *Bifidobacterial* populations were found in pediatric IBD patients (51). *Bifidobacterium animalis* supsp. *lactis* was proved to have protective capacity on acute and chronic colitis in mice (52). In our study, a positive spearman correlation between *Bifidobacterium animalis* and γδT17 cells was detected, which enhanced the important regulatory role of *Bifidobacteria* on colon mucosa. In addition, we also detected a positive correlation between *Bacillus* species with γδT17 cells. The *Bacillus* species has also been demonstrated to ameliorate DSS-induced dysbiosis and gut inflammation by balancing beneficial and harmful bacteria and associated anti- and pro-inflammatory agents (53). However, when tested *in vitro*, we found that only the *Bacillus* strain, but not the *Bifidobacterium* strain, showed promoting effects on TLR2 expression and IL-17A secretion by γδT cells, which suggested that some bacteria in the consortium may contribute to the expansion of intestinal γδT17 cells, and bacteria such as *Bifidobacterium* spp. may benefit from the re-established balance between gut microbiota and intestinal immune system and, in turn, exert beneficial effects on colon mucosa.

In conclusion, our results suggest that BCT can restore the alliance between commensal microbiota and intestinal γδT17 cells through the manipulation of intestinal dysbiosis in DSS-treated mice and this contributes to the improvement of mucosal barrier function, which is closely associated with the upregulation of the IL-17A-Act1-occludin regulatory pathway. Bacteria, such as *Bacillus* and *Bifidobacterium*, may be responsible for or affected by the upregulation of γδT17 cells. However, further study is needed to clarify the underlying mechanisms.

## Ethics Statement

The animal experimental procedures were approved by the Medical Ethics Committee of Dalian Medical University, China (SYXK2015-0002). The strains used for bacterial transplantation are deposited in the Bacteria Collection of Dalian medical University (DMBC), China, and The University’s Institutional Biosafety Committee (IBC) approved applications to conduct only scientific research with these microorganisms.

## Author Contributions

XL and JY designed the research; ML, BW, XS, YT, XW, BG, YT, YD, and CH performed the experiments; ML and BW analyzed the data; ML and XL wrote the manuscript. All authors read and approved the final manuscript.

## Conflict of Interest Statement

The authors declare that the research was conducted in the absence of any commercial or financial relationships that could be construed as a potential conflict of interest.

## Supplementary Material

Figure S1 The composition of bacterial composition in different experimental groups at Class and Order levels.

Figure S2 Heatmaps of the abundance of the dominant Phyla **(A)**, Family **(B)**, and Genera **(C)** in different groups. The relative abundance of each microbial group was normalized, and the *Z*-value was presented and depicted by the color intensity.

Figure S3 The specific bacterial groups that are significantly manipulated by BCT. The MetaStat method was used to identify the significantly reduced or elevated microbial groups by DSS treatment and was reversed by BCT. k, kingdom; p, phylum; c, class; o, order; f, family; g, genus; All values are mean ± SEM (*n* = 5). *adjusted *p* value <0.05; **adjusted *p* value <0.01.
