# Supplementary material for: Upregulation of Intestinal Barrier Function in Mice with DSS-Induced Colitis by a Defined Bacterial Consortium Is Associated with Expansion of IL-17A Producing Gamma Delta T Cells
Source: Front Immunol. 2017 Jul 12;8:824. doi: 10.3389/fimmu.2017.00824 (PMC5506203; doi:10.3389/fimmu.2017.00824)
Supplement: Supplementary file 1 [file Data_Sheet_1.DOCX]

**Table S1. Bacterial strains and their population in the bacterial consortium used in this study.**

| **Strain** | **Strain with highest identity** | **Identity**  **(%)** | **Phylum** | **Gram stain** | **Population in the consortium (LogCFU/ml)** |
| --- | --- | --- | --- | --- | --- |
| DMBCT1 | *Bifidobacterium* *thermophilum* RBL67 | 98 | *Actinobacteria* | + | 8.523 |
| DMBCT2 | *Escherichia coli* str. K-12 | 99 | *Proteobacteria* | - | 6.012 |
| DMBCT4 | *Staphylococcus* *aureus* subsp. NCTC 8325 | 99 | *Firmicutes* | + | 7.321 |
| DMBCT5 | *Fusobacterium nucleatum* subsp. vincentii _1_36A2 | 97 | *Fusobacteria* | - | 7.372 |
| DMBCT6 | *Lactobacillus* *reuteri* DSM 20016 | 99 | *Firmicutes* | + | 9.145 |
| DMBCT7 | *Bacteroide* *salanitronis* DSM 18170 | 94 | *Bacteroidetes* | - | 7.214 |
| DMBCT8 | *Streptococcus* *thermophilus* CNRZ1066 | 98 | *Firmicutes* | + | 9.225 |
| DMBCT9 | *Veillonella* *parvula* DSM 2008 | 98 | *Firmicutes* | - | 8.384 |
| DMBCT10 | *Peptococcus niger* spp. | 94 | *Firmicutes* | + | 4.449 |
| DMBCT11 | *Eubacterium* *siraeum* V10Sc8a | 94 | *Firmicutes* | + | 7.087 |

**Table S2. Primers used for quantitative real-time PCR analysis.**

| **Primer mane** | **Sequences of primers** | **Tm (°C)** | **References** |
| --- | --- | --- | --- |
| *Cldn1* | Forward: 5'-AAG ACG ATG AGG TGC AGA AG-3'  Reverse:5'-GTG AAG AGA GCC TGA CCA AA-3' | 65 | [1] |
| *Ocln* | Forward: 5'-GTACCCACCAGTGACCAACA-3'  Reverse:5'-GTTGCTGGAGCTTAGCCTGT-3' | 65 | [1] |
| *il17a* | Forward: 5'-CAGCAGCGATCATCCCTCAAAG-3'  Reverse:5'-CAGGACCAGGATCTCTTGCTG-3' | 55 | [2] |
| *RORγt* | Forward: 5'-CCGCTGAGAGGGCTTCA-3'  Reverse:5'-TGCAGGAGTAGGCCACATTACA-3' | 55 | [2] |
| *GADPH* | Forward: 5'-ACCACAGTCCATGCCATCAC-3'  Reverse: 5'-TCCACCACCCTGTTGCTGTA-3' | 55 | [3] |

1. Ahn C, Shin DH, Lee D, Kang SM, Seok JH, Kang HY, et al. Expression of claudins, occludin, junction adhesion molecule A and zona occludens 1 in canine organs. *Mol Med Rep* (2016) **14**, 3697-7032016. doi: 10.3892/mmr.2016.5725.
2. Ye L, Wen Z, Chen B, Yu T, Liu L, Zhang J, *et al.* Interleukin-10 attenuation of collagen-induced arthritis is associated with suppression of interleukin-17 and retinoid-related orphan receptor γt production in macrophages and repression of classically activated macrophages. *Arthritis Res Ther* (2014) **16**, R96. doi: 10.1186/ar4544.
3. Liu G, Guo J, Liu J, Wang Z, Liang D*.* Toll-like receptor signaling directly increases functional IL-17RA expression in neuroglial cells. *Clin Immunol* (2014) **154**, 127-140. doi: 10.1016/j.clim.2014.07.006.
