# Supplementary figures and images for: Upregulation of Intestinal Barrier Function in Mice with DSS-Induced Colitis by a Defined Bacterial Consortium Is Associated with Expansion of IL-17A Producing Gamma Delta T Cells
Source: Front Immunol. 2017 Jul 12;8:824. doi: 10.3389/fimmu.2017.00824 (PMC5506203; doi:10.3389/fimmu.2017.00824)

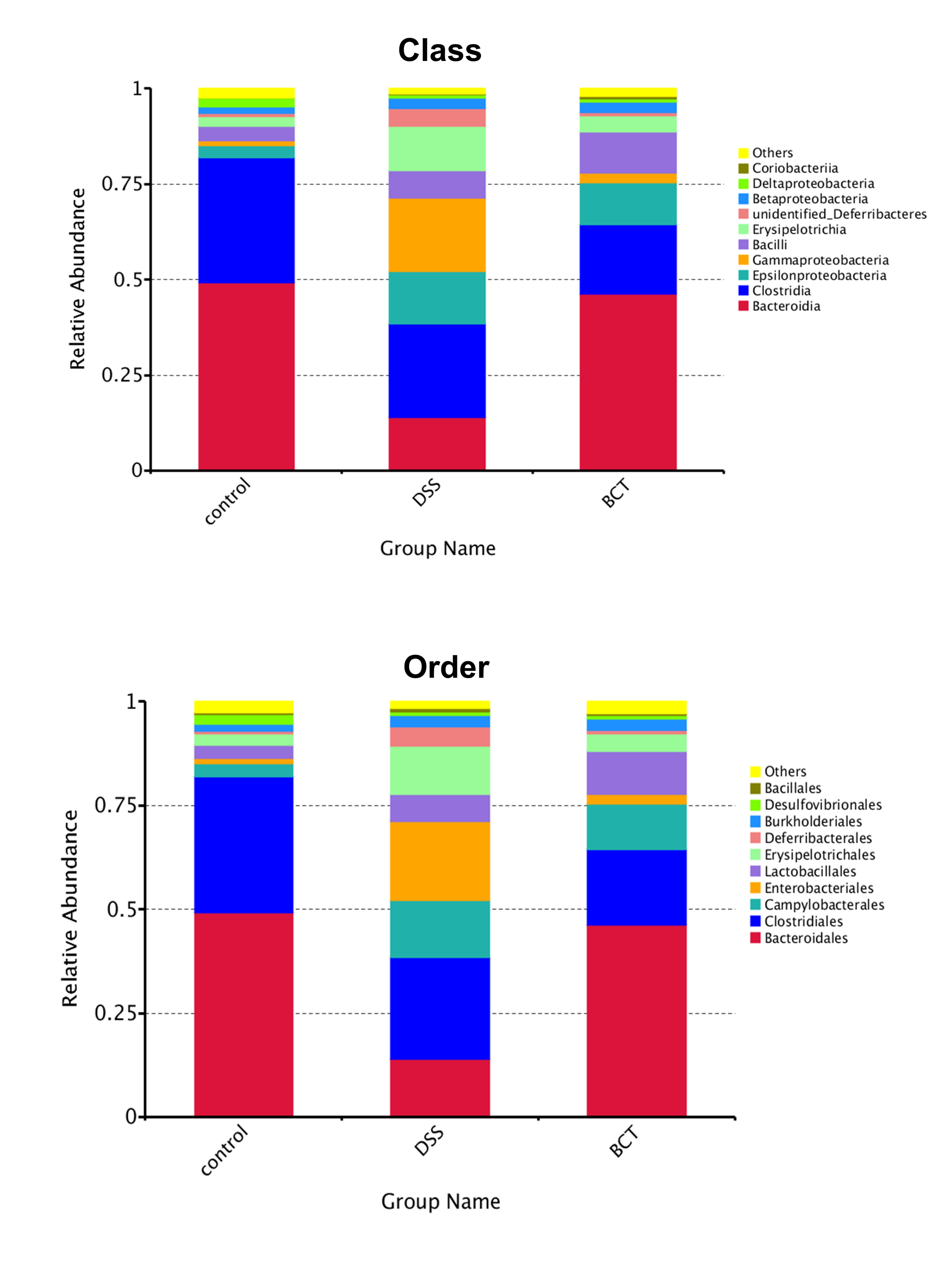

Supplement: Figure S1 — The composition of bacterial composition in different experimental groups at Class and Order levels. [file Image_1.TIF]

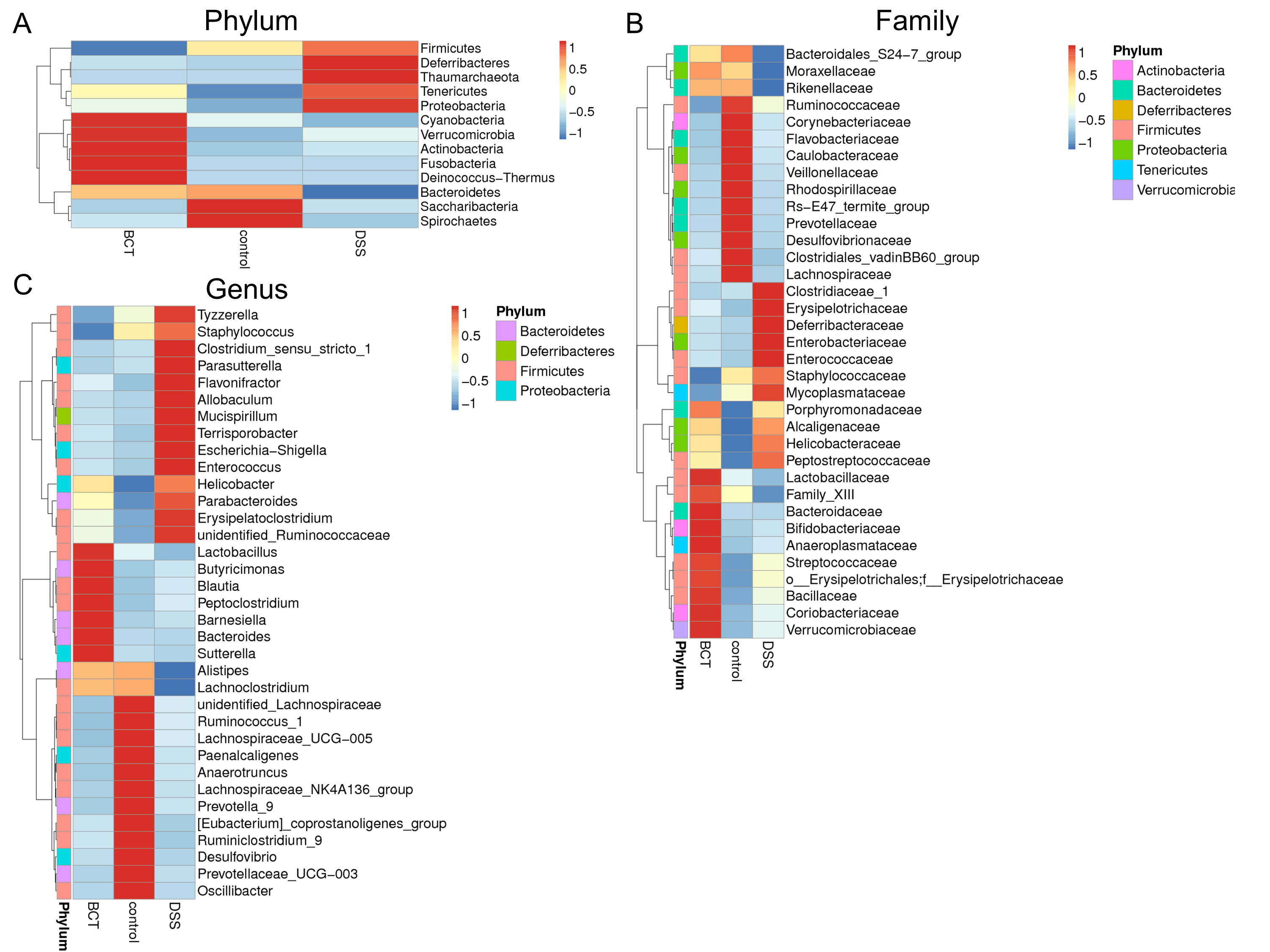

Supplement: Figure S2 — Heatmaps of the abundance of the dominant Phyla (A), Family (B), and Genera (C) in different groups. The relative abundance of each microbial group was normalized, and the Z-value was presented and depicted by the color intensity. [file Image_2.TIF]

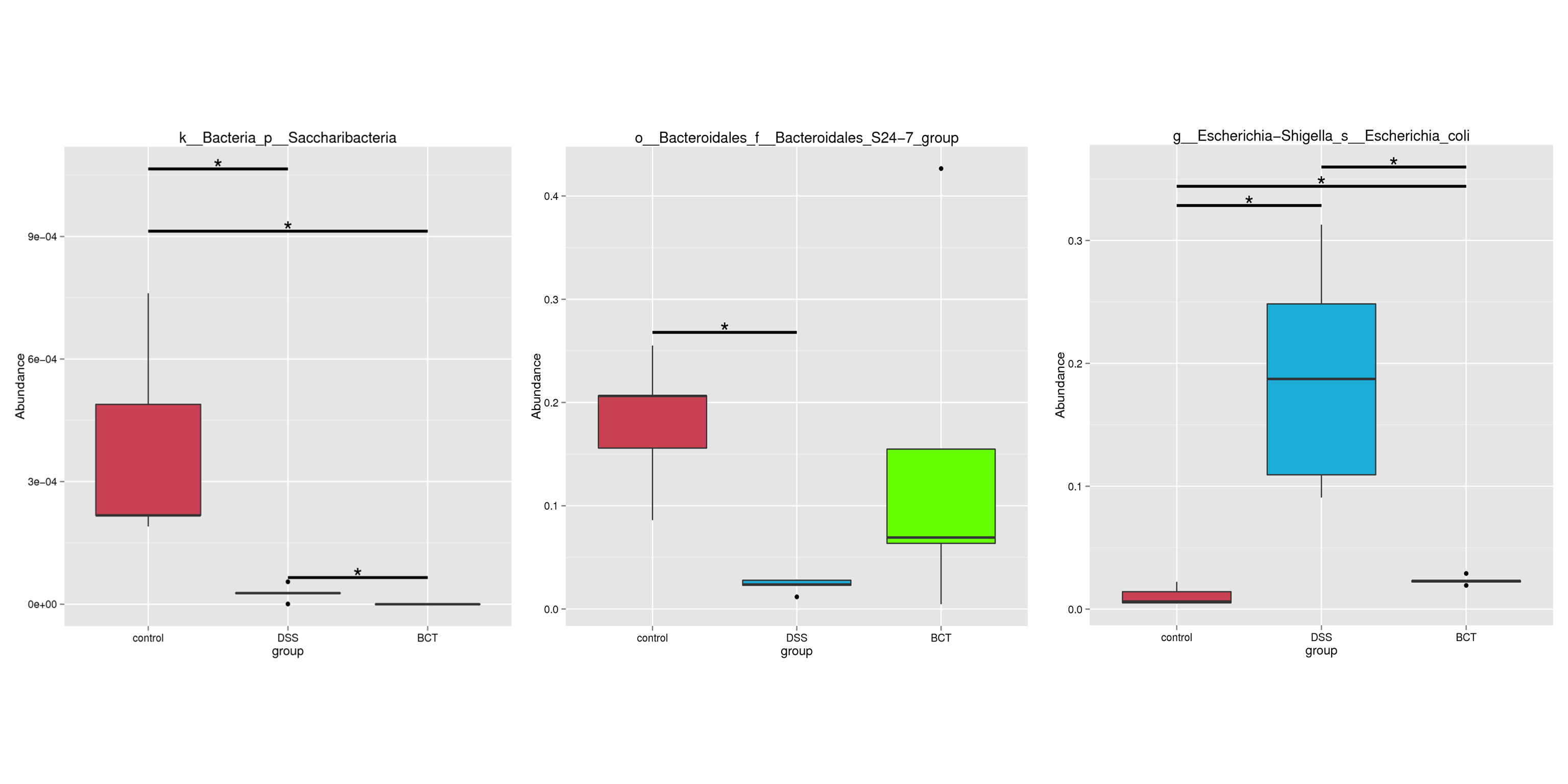

Supplement: Figure S3 — The specific bacterial groups that are significantly manipulated by BCT. The MetaStat method was used to identify the significantly reduced or elevated microbial groups by DSS treatment and was reversed by BCT. k, kingdom; p, phylum; c, class; o, order; f, family; g, genus; All values are mean ± SEM (n = 5). *adjusted p value <0.05; **adjusted p value <0.01. [file Image_3.TIF]
